# Sustainable Agriculture Systems in Vegetable Production Using Chitin and Chitosan as Plant Biostimulants

**DOI:** 10.3390/biom11060819

**Published:** 2021-05-31

**Authors:** Mohamad Hesam Shahrajabian, Christina Chaski, Nikolaos Polyzos, Nikolaos Tzortzakis, Spyridon A. Petropoulos

**Affiliations:** 1 Department of Agriculture, Crop Production and Rural Environment, University of Thessaly, Fytokou Street, 38446 Volos, Greece; cchaski@uth.gr (C.C.); npolyzos@uth.gr (N.P.); 2 Department of Agricultural Sciences, Biotechnology and Food Science, Cyprus University of Technology, 3603 Limassol, Cyprus; nikolaos.tzortzakis@cut.ac.cy

**Keywords:** chitosan derivatives, oligochitosan, vegetable crops, abiotic stress, biotic stress

## Abstract

Chitin and chitosan are natural compounds that are biodegradable and nontoxic and have gained noticeable attention due to their effective contribution to increased yield and agro-environmental sustainability. Several effects have been reported for chitosan application in plants. Particularly, it can be used in plant defense systems against biological and environmental stress conditions and as a plant growth promoter—it can increase stomatal conductance and reduce transpiration or be applied as a coating material in seeds. Moreover, it can be effective in promoting chitinolytic microorganisms and prolonging storage life through post-harvest treatments, or benefit nutrient delivery to plants since it may prevent leaching and improve slow release of nutrients in fertilizers. Finally, it can remediate polluted soils through the removal of cationic and anionic heavy metals and the improvement of soil properties. On the other hand, chitin also has many beneficial effects such as plant growth promotion, improved plant nutrition and ability to modulate and improve plants’ resistance to abiotic and biotic stressors. The present review presents a literature overview regarding the effects of chitin, chitosan and derivatives on horticultural crops, highlighting their important role in modern sustainable crop production; the main limitations as well as the future prospects of applications of this particular biostimulant category are also presented.

## 1. Introduction

Modern agriculture needs to be adapted to the ongoing climate change and the growing food demands due to increasing population. Considering the finite natural resources, sustainable cropping is of major importance, especially in horticultural crops that are more susceptible to climate extremities and more demanding in terms of agricultural inputs [1,2]. In this context, biostimulant application is considered a novel, eco-friendly farming practice that marries two otherwise contrasting concepts, namely crop intensification and sustainability [3,4]. So far, biostimulant products form a significant part of the global farming industry, showing increasing trends over the years and in the years to come [5]. There are numerous reports regarding their positive effects on crops, especially under biotic and abiotic stress conditions [6,7,8], while significant research is continuously conducted to find and/or produce new biostimulatory products [9,10,11], as well as to reveal the mechanisms of action behind the observed effects [12,13,14]. However, the variability in the composition of biostimulant products, as well as the lack of common application protocols for the various products, may create inconsistencies between the observed results and complicate the efforts to reveal the actual mechanisms behind the biostimulatory effects, which may include physiological processes, morphological changes and hormonal regulation [12,14,15,16].

Biostimulants’ beneficial activity involves the induction of root growth, the improvement in nutrients uptake and the production of phytohormones, while osmotic adjustment via synthesis of organic osmolytes has been also confirmed [17,18,19,20]. Biostimulants also can be used to reduce application of mineral inorganic fertilizers and are being considered an environmental friendly practice with no significant adverse impacts on both fruit quality and total yield [21,22,23,24]. Humic acids, fulvic acid, protein hydrolysates, seaweed extracts, *N*-containing compounds, botanicals, seaweed extracts, chitosan and other related biopolymers, beneficial bacteria and fungi and inorganic compounds are the main categories of plant biostimulants [25,26,27]. However, different classification approaches have been suggested so far, based either on the origin of each biostimulant, namely biological or non-biological, microbial and non-microbial, or on the mode of action which divides biostimulants into phytohormonal and non-phytohormonal ones [28].

Modern crop production has to cope with biotic and abiotic stressors such as soil and irrigation water salinity, water limitations, extreme and untimely weather phenomena and infections from pathogens and pests, which severely affect crop performance and quality of the final products [29,30]. In this context, the application of chitin, chitosan and derived biopolymers can play a pivotal role due to their confirmed biostimulatory activity in various crops, especially in vegetable species, which are more prone to stressors [31,32,33]. Different sources of chitin and chitosan in nature are Crustaceans (shrimp, lobster, king crab), Fungi (*Mucor rouxii*, *Aspergillus niger*, *Penicillum crysogenum*, *Lactarius vellereus*), Insects (lady bug, silk worm, wax worm, butterfly) and mollusks (shell oysters, squid pen) [34]. Crustacean shells are the most notable chitin source, and chitin recovery involves three steps consisting of demineralization, deproteination and elimination of pigments and lipids [35,36]. Microbial proteases such as *Bacillus* sp., *Lactobacillus* sp., *Pseudomonas* sp., *Serrati marcescens*, etc. are the most notable applied strains of chitin and chitosan production [35]. 

Economic indicators such as return on investment, net present value and payback period have been reported as important characteristics for a mass integrated biorefinery approach to produce chitin and chitosan [36]. Considering the great amounts of chitinous waste production (e.g., 2.1–2.7 Mt in 2011), there is great economic potential of finding alternative uses of chitin and valorizing biowaste [37]. Various application have been suggested for chitin obtained mostly from crustacean shells, which are also a very good source for carotenoids recovery (e.g., astaxanthin) [38]. Due to its biological and physicochemical properties, the most important applications of chitin and its derivatives are in (a) food application, due to chitosan’s ability in lowering cholesterol by blocking the absorption of cholesterols and dietary fat, which facilitates weight and body fat loss in the human body [39], controls over-nutrition and achieves insulin resistance therapy [40,41]; and (b) biomedical application, having tremendous biological benefits such as biodegradation, biocompatibility, anticancer, antibacterial, non-toxicity, immune-stimulating effects, hemostatic activity in cell culture, wound healing, tissue engineering and drug delivery [42]. Chitooligosaccharides and their derivatives are the appropriate agents capable of treating or preventing various chronic inflammation such colitis, hepatitis, gastritis, periodontal disease and through drug delivery systems [43,44,45]; (c) agricultural applications [46,47,48], and (d) bionanotechnology, such as the versatile potential uses in cosmetics, photography, ophthalmology, textile industry and water and waste treatment [49,50]. It has been also reported that large-scale chitosan commercialization originates from the chemical alkaline hydrolysis of shrimp chitin, with a cost of nearly USD 10/g (Sigma Chemical Con., St. Louis, MO 63118, USA) [51], but agro-industrial wastewaters have been also used as alternative media for fungi grown in submerged fermentation, which are readily available and have a low cost to use, saving around 38–73% of the total cost of the bioproduct production [51,52]. However, cost production is flexible since it includes transportation and labor costs, which vary significantly around the world [38].

Therefore, the present review provides an overview of the recent trends in biostimulant application focusing on the effects of chitin, chitosan and derivatives on the main vegetable crops, as well as on the main mechanisms of action. Finally, the main limitations and the needs for future research will be presented. 

## 2. Methods of Obtaining Chitin and Chitosan Used in Agricultural Production as Biostimulants

Chitin and chitosan are produced by two major extraction methods, namely chemical and biotechnological. Chemical processes are based on the use of strong acids and bases are currently the most widely applied methods in both laboratory and industrial scale production [53]. Two well-known methods of chitosan production are to extract chitosan directly from cell walls of molds, and thermo-chemical or enzymatic methods of chitin deacetylation to remove the N-acetyl groups from chitin. At present, chitosan is manufactured industrially through thermo-chemical hydrolysis of chitin’s amide bonds [53]. Several forms such as solutions, flakes, fine powder, beads and fibers are available for commercial preparations of chitosan [54]. Chitooligosacchrides can be produced through chemical, physical, electrochemical and enzymatic degradation of chitin and chitosan [53]. The most commonly applied chemical methods of chitooligosaccharides production include acid degradation and oxidation degradation of chitin and chitosan [53]. 

The traditional systems for commercial preparation of chitosan from various sources may lead to some drawbacks and many disadvantages since they are not cheap or environmentally friendly, and have inconsistent molecular weight and degree of acetylation [55,56]. A promising economical method for innumerable application and the production of highly viscous chitosan is the use of biotechnology fermentation processes, such as deproteination and demineralization by organic acid bacteria and protease and deacetylation by chitin deacetylase [56,57]. Chitosan can be promoted as a green product [35], and chitosan from crustacean as a food industry waste is economically feasible [58,59,60]. Although chitosan is mainly obtained from crustacean shells rather than from insect and fungal sources, the commercialization of chitosan extraction from insect and fungal sources has increased in recent years [35]. Techno-economic sensitive approaches have also been performed for chitosan production from shrimp shell wastes [61]. 

The chemical methods for production of chitin and their derivatives that are currently being applied on a commercial scale consist of two steps, namely, deproteinization by alkali treatment and demineralization by acidic treatment under high temperature, followed by the decolorization step which focuses on removing lipids and pigments [35]. Ambient temperature and stirred bioreactors have been applied to improve the quality and to shorten the process [50,62]. Crustacean wastes from the shrimp and crab industry are pretreated with washing and grinding, and then the grinded exoskeleton goes through depigmentation by ethanol. After that, the exoskeleton proceeds to the demineralization stage by hydrochloric acid, and then the exoskeleton proceeds to the deproteinization stage by sodium hydroxide and provides chitin. Finally, chitin goes through deacetylation by sodium hydroxide and produces chitosan [61,63]. 

## 3. Practical Applications of Chitosan on Vegetable Crops

Chitosan is an environmentally and eco-friendly polymer with multipurpose applications in various fields such as agriculture, cosmetology, food, paper, pharmacy and textile industries [64,65,66,67,68] and a potent agent for the removal of toxic pollutants [69,70]. It can be used in plant production systems as a single compound or combined with other polymers and elements [68,71]. It is considered one of the most abundant natural biopolymers. It is derived from chitin and its structure consists of two sub-units, namely D-glucosamine and N-acetyl-D-glucosamine, connected with 1,4-glycosidic bonds to each other [72,73]. Its ability to bind on other compounds allows the delivery of nutrients, pesticides and biomolecules in plants systems [71,74,75]. The precursor of chitosan (chitin) is the second most renewable source of carbon throughout the world, which makes chitosan a very promising material for industrial applications, with more than 2000 tons produced annually [76]. However, the preparation of chitosan via industrial methods produces a final product that cannot be described accurately as chitosan since it contains various polymers with different degrees of polymerization and physical properties [72,77]. Despite this downside, the benefits from chitosan application are far more important since it is claimed to be GRAS (generally recognized as safe) and easily absorbed, inexpensive, easily available and easy to manipulate [78,79].

The beneficial activities of chitosan are mostly associated with increased photosynthetic activity, tolerance to abiotic stressors such as drought, salinity and extreme temperatures, as well as with increased antioxidant enzymes activity and the expression of defensive genes [80]. There are numerous examples of chitosan application on vegetable crops; however, the obtained results are not always consistent since the various studies differ in their methodological approach (time and dose of application), while chitosan-based biostimulant products may also differ in chemical composition and chitosan content, which further increases heterogeneity in biological effects [77,78,81,82,83]. The primary use of chitosan in agriculture is based on its eliciting effects on the biosynthesis of protective biomolecules against pests and pathogens [14,84,85], as well as on the up-regulation of defensive genes [86,87]. 

It can be applied in various forms including seed coating, foliar spraying or soil incorporation and as a coating agent in fruit and vegetables for post-harvest protection [31,88,89]. The main activity of this biopolymer is plant protection against various biotic and abiotic stressors via various mechanisms that must be unraveled. For example, the hydrophilic nature of chitosan may alleviate stress effects by reducing water content in cells [14], while it can also increase root length and reduce the transpiration rate, resulting in improved water uptake and water use efficiency in plants [90,91]. Moreover, chitosan application may result in plant growth improvement mostly through the increased nitrogen and nutrients uptake, while it can be used as an extra carbon source in plant biosynthetic processes [82,92]. Other activities include the effects of mycorrhization in tomato plants through the regulation of the expression of endochitinase-encoding genes [81]. Moreover, the foliar application of chitosan may serve as a physical barrier against pathogens [93], while it can increase the thickness of cell walls in the leaves’ epidermis, contributing to tolerance against pathogens attacks [94]. Its use as soil amendment has also found practical applications in agriculture, resulting in increased yield in lettuce [95] and tomato [96] crops, while it can remediate polluted soils through the removal of cationic and anionic heavy metals and the improvement of soil properties [97,98,99]. Seed coating with chitosan may increase germination percentage and seedling growth through the induction of antioxidant enzymes [100,101,102], or the increased water absorption through the formation of a semi-permeable coating on the seed surface [103,104]. Finally, coating fresh fruit and vegetables with chitosan may increase shelf life [105], retain the quality and prevent spoilage from food-borne pathogens [106,107,108] and microbes that affect human health [109]. 

Apart from the direct effects of chitosan, there are several applications of chitosan derivatives and nanoparticles, which are used as carriers of nutrients and other compounds. Chitosan-based biodegradable nanomaterials (NMs) consist of nanogels, nanospheres, nanocapsules, nanoparticles and nanocomposites, which have been applied for plant growth promotion and plant protection especially against viruses, fungi and bacteria, providing a new and effective tool for sustainable crop protection [7,110,111]. For example, chitosan-coupled copper nanoparticles (ch-CuNPs) have several advantages as a growth promoter and fungicide, showing promising properties for substituting conventional pesticides and ameliorating their hazardous impacts on the environment [112]. Chitosan nanoparticles were also suggested to increase immunity against pathogens through the induction of innate defense mechanisms and defense-related enzymes [97]. Moreover, the application of nanochitosan solutions via soaking of seedlings or foliar spraying showed better results in terms of onion crop performance and nutrient use efficiency [113]. The advanced techniques in nanoparticle preparation have allowed the efficient capitalization of chitosan’s beneficial effects not only in agriculture but also in the food industry through functional packaging [67,68,114]. The use of bio-nanomaterials may also find uses in smart genetic engineering in plants through the editing of plant genomes [71]. However, the use of such materials for human-related purposes is currently restricted and under debate and further studies are needed to transfer the achieved knowledge from the laboratory to an industrial scale and to manifest the positive effects with large-scale trials [71,115,116]. 

The most notable impacts of chitosan on various vegetable plants are presented in Table 1. 

## 4. Activities and Applications of Oligochitosan

Oligochitosan (or chitooligosaccharides), with 3 to 10 saccharide residues of N-acetylglucosamine or glucosamine, include homo or hetero oligomers obtained from chitin by chemical or enzymatic hydrolysis [145], or though oxidative and ultrasonic degradation [146,147]. It is considered a plant elicitor and has similar effects as chitosan on plants against biotic stress and plant growth improvement [148], while it also possesses significant beneficial properties for human health [149]. Moreover, similarly to chitosan, the biological activities of oligochitosan are also dependent on the degree of polymerization (DP) and the acetylation pattern, as well as on the concentration of the applied compound and the plant species [46]. In particular, oligochitosan with higher DP showed stronger elicitation effects through the expression of defensive genes [150]. 

The main activities of oligochitosan are associated to the induction of secondary metabolites biosynthesis and the activation of plant innate immunity through signal perception and transduction, expression of defensive genes and finally the accumulation of protective secondary metabolites [151,152]. So far, most of the applications refer to field crops and a limited number of studies evaluated the effects of oligochitosan on vegetable crops. For example, oligochitosan application showed higher in vitro effectiveness than chitosan in inhibiting mycelia growth of *Phytophthora* species [153,154], while it promoted plant growth and yield in various vegetable crops such as common bean, potato, tomato, chili pepper, spinach and eggplant [155,156,157,158]. The combined application of oligochitosan and ε-poly-L-lysine in tomato plants showed synergistic effects against *Botrytis cinerea* infections both under in vitro and in vivo conditions, suggesting their use as a bio-fungicide alternative to synthetic fungicides [159]. Moreover, Li et al. [160] suggested that oligochitosan induced the production of nitric oxide and hydrogen peroxide in *Brassica napus* L. plants, which acted as signaling molecules in the regulation of stomata closure and the expression of *LEA* protein gene for the protection against drought. Apart from protective effects, oligochitosan may improve the functional properties of vegetable products, as already reported in case of white radish sprouts (*Raphanus sativus* L.) where seed germination with oligochitosan-treated water resulted in a significant increase in the most abundant glucosinolate, e.g., glucoraphasatin [161]. 

The use of oligochitosan has great potential for farming applications, especially in crops with high added value as in the case of vegetable species. However, future research is needed to define important parameters regarding the biostimulant product, such as the degree of polymerization, and fine tune the application practices related to dose and application time and method. 

## 5. The Use of Chitin as Biostimulant

Chitin is a versatile polymer of *β*-1,4-*N*-acetylglucosamine widely abundant in nature, and mainly obtained from prawn/crab shells for commercial purposes [38,72,162], while the isolation of chitin from edible fungi production chain has also been considered [163,164]. It is composed after the polymerization of *N*-acetylglucosamine through the activity of chitin synthases which are classified in three divisions and seven classes [165]. Chitin is the second most abundant polysaccharide in living organisms after cellulose, being the main structural compound in fungal cells and the skeleton of invertebrates [38,166]. The main differences of chitosan are its hydrophobic nature and the lower solubility in water and several organic solvents, which pose restrictions in practical applications in agriculture and significantly affect the biological properties of chitin [167]. Therefore, its chemical modification and the derivatives obtained through chemical reactions are of major importance towards the better exploitation and valorization of this biopolymer. The current market trends show that the global chitin market is expected to reach USD 2900 million by 2027, with healthcare, waste and water treatment and agrochemicals sectors being the largest market segments [168]. 

The main application methods of chitin on plants consist of foliar spraying and direct soil application, while it may be also applied on coating horticultural products to increase their shelf life after processing [169,170,171]. When foliar spraying is applied, the positive effects of chitin are associated with its direct act as a physical barrier against pathogen infections or with indirect activities that induce the plant immune system as signaling molecules for defense pathways [167,172,173]. Soil application effects are more complex than foliar spraying and include (a) the increased bioavailability of nitrogen due to the high content of chitin in this important macronutrient and the low C/N ratio [174], (b) the increased activity of chinolytic organisms that may have antagonistic effects against plant soil pathogens [175] and (c) the favorable effects on soil microbiota such as mycorrhiza and rhizobia that act synergistically to plant and improve crop performance [176,177]. On the other hand, the use of chitin in edible coatings of horticultural products may provide a semipermeable physical barrier that regulates gas exchange and may delay ripening and decrease water losses and respiration rates [178]. However, these effects may differ since chitin is a natural product and differences in composition and physicochemical properties (e.g., nitrogen and ash content, degree of deacetylation, bulk density and viscosity) of commercial products may result in differences in biological activities [179]. 

Several studies have evaluated the biostimulatory effects of chitin on vegetables crops. For example, Rajkumar et al. [85] suggested that combining chitin with salicylic acid may increase the population of *Pseudomonas* sp. strains SE21 and RD41 which act antagonistically against *Rhizoctonia solani,* causing damping off in pepper plants. Moreover, chitin obtained from yeast cell walls may increase the tolerance of tomato fruit against *Botrytis cinerea* [180]. Peat supplementation with chitinolytic plant growth promoting *Bacillus subtilis* AF1 resulted in increased emergence and plant growth of pigeon pea seedlings [181], while the amendment of peat substrate with chitin increased the rhizobiome of lettuce, resulting in improved plant growth [182]. Chitin has also been used in biocontrol agents against soil-borne and foliar plant pathogens and pests [183]. Considering the disadvantageous properties of chitin that limit its direct application in plants, complex structures have been suggested such as the protein/CaCO_3_/chitin nanofiber complex which improved plant growth in hydroponically grown tomatoes [184], or polymeric chitin nanofibers which exhibit eliciting activities [185]. Chitin nanofibers were also effective in inhibiting the infections by *Alternaria brassicicola* and *Colletotrichum fructicola* in cabbage and strawberry plants, respectively [186], as well as in increasing the tolerance against *Fusarium* wilt [187] or improving nitrogen use efficiency and promoting the growth of tomato plants [188]. Moreover, the application of a formulation based on chitin and *Trichoderma* ameliorated the occurrence of head rot (*Sclerotinia sclerotiorum* (Lib.) deBary) and root-knot (*Meloidogyne incognita* Kofoid and White; Chitwood), while it also increased the yield of cabbage plants grown under field conditions [189]. The application of betaine and chitin in lettuce plants grown under a regulated water deficit irrigation regime increased crop performance, as expressed by improved water use efficiency values [190]. Chitin oligosaccharide dithicyclobutane derivative showed nematicidal activity against *Meloidogyne incognita* in tomato seedlings, an effect that could be associated with its glutathione binding activity [191]. 

Table 2 presents the most important effects of chitin application on vegetable crops.

**Figure 1:**
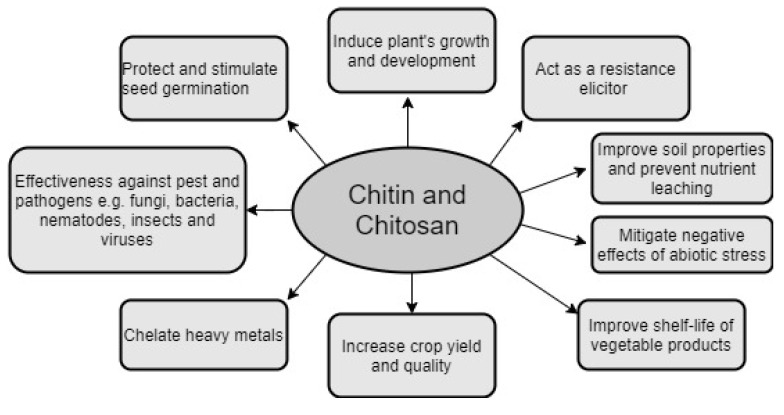
The most important effects of chitin and its derivatives’ applications.

**Figure 2:**
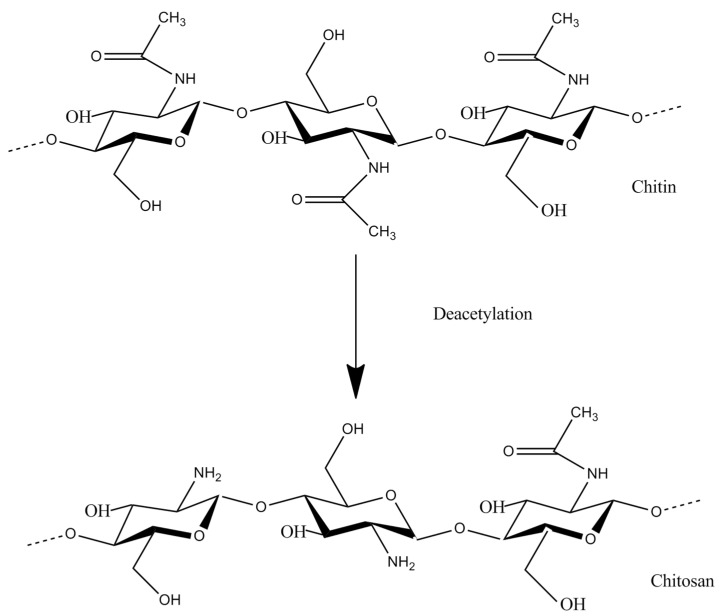
The chemical structure of chitin (C_8_H_15_NO_6_) and chitosan (C_56_H_103_N_9_O_39_).

Figure 1 shows the most notable advantages of chitin and chitin derivatives application, while the chemical structures of chitin (C_8_H_15_NO_6_) and chitosan (C_56_H_103_N_9_O_30_) are shown in Figure 2.

## 6. Conclusions

Chitin, chitosan and chitosan oligosaccharides are natural biopolymers with numerous activities in plants. So far, the practical applications of these compounds have shown beneficial effects on the protection of horticultural plants against pathogens, and on plant productivity and growth, especially under environmental constraints which highlight its promising roles for crop cultivation under drought conditions in arid and semi-arid regions. Among these compounds, chitosan seems to be the most economical option for improving productivity and quality of various plants at the moment, especially in high added value species such as horticultural crops. On the other hand, chitin single applications are limited mostly due to practical limitations related to the hydrophobic and insoluble nature of this compound. Therefore, various transformations must be considered in order to valorize its beneficial effects on plants through the complexation with other compounds or the nanofibrillation. Moreover, the diverse sources of chitin and derivatives make it difficult to standardize the composition of commercial products, which may affect their biological activities in plants. Finally, the farming sector is a very promising alternative to exploit these natural biopolymers and provide farmers a sustainable tool to increase crop productivity and the quality of the final product. However, further studies are needed to improve reproducibility of the positive effects and to standardize the production processes from the lab to an industrial scale. Both of these aspects will help towards improving the application protocols of biostimulant products with standardized composition.

## Figures and Tables

**Table 1:** The effects of chitosan on vegetable crops.

Plant	Scientific Name	Plant Family	Key Point	Reference
Artichoke	*Cynara scolymus* L.	Asteraceae	Chitosan application promoted germination and plant growth of artichoke and induced a significant decrease in fungi infections.	[117]
Basil	*Ocimum basilicum* L.	Lamiaceae	Chitosan may ameliorate harmful impacts of drought on basil growth, as well as improve total phenol and antioxidant activity.	[92]
			Chitosan lactate foliar application may promote the accumulation of bioactive substances; increase the activity of antioxidant enzymes; improve photosynthetic rate and plant growth.	[118]
Bean	*Phaseolus vulgaris* L.	Fabaceae	Chitosan increased the yield on normal or delayed sowing.	[119]
			Effective impact of nanocarriers composed alginate/chitosan (ALG/CS) in promoting leaf area and the levels of chlorophylls and carotenoids.	[120]
Bell pepper	*Capsicum annuum* L. var. *grossum* (L.) Sendt	Solanaceae	Chitosan nanoparticles (CsNPs) indicated the significant role in anti-biofilm activity against foodborne pathogens.	[121]
			Chitosan nano-coating (CsNC) lengthened the shelf life of fresh-cut bell pepper.	[121]
			Chitosan treatments increased germination, improved seedling growth and emergence in cold test.	[122]
			CaCl_2_-tea tree oil (TTO)/low molecular weight chitosan (LMWCS) slowed down the microbial growth in fresh-cut bell pepper.	[114]
Chickpea	*Cicer arietinum* L.	Fabaceae	Chitosan nanoparticles-loaded application with thiamine increased germination percentage and growth in chickpea.	[123]
			Foliar application stimulated protection of chickpea seedlings against wilt disease, and increased indole acetic acid (IAA) production in seedlings.	[124]
Chilli	*Capsicum frutescence* L.	Solanaceae	Chitosan applied as seed treatment (1%) and foliar spray (0.5%) combined application showed the highest effectiveness in controlling anthracnose of chili and stimulated yield and yield contributing characters.	[125]
Cucumber	*Cucumis sativus* L.	Cucurbitaceae	Chitosan seed treatment resulted in 100% resistance against damping off caused by *Phytophthora capsici*.	[126]
			Chitosan may synthesize defense-responsive enzymes and stimulate phytohormones in cucumber plants.	[102]
Eggplant	*Solanum melongena* L.	Solanaceae	The synthesized nanocomposites improved both the nematocidal activity and the plant systematic immune response.	[127]
Faba bean	*Vicia faba* L.	Fabaceae	The new carboxymethyl chitosan-titania nanobiocomposites may decrease negative effects of *Bean yellow mosaic virus* (BYMV).	[128]
Ginger	*Zingiber officinale* Roscoe	Zingiberaceae	Chitosan and oligochitosan suppressed ginger rhizome rot in storage.	[129]
			Chitosan and oligochitosan improved defense enzymes activity in ginger.	[129]
Lettuce	*Lactuca sativa* L.	Asteraceae	Chitosan application at 2% in a Ni-contaminated soil may significantly regulate Ni bioavailability.	[130]
			Chitosan nanoparticles (CNPs) loaded with indole-3-acetic acid (IAA) indicated a beneficial impact on the hydroponic lettuce growth.	[131]
Onion	*Allium cepa* L.	Amaryllidaceae	Nano chitosan may improve the efficiency of traditional fertilizers and promoted the net return per fed.	[113]
			Chitosan/polyacrylic acid hydrogel nanoparticles (CS/PAA-HNPs) stimulated the yield, plant growth and nutrient content in onion bulbs.	[132]
Okra	*Hibiscus esculentus* L.	Malvaceae	Chitosan foliar application at 100 or 125 ppm may be applied at early growth stages to achieve higher yields.	[133]
Pea	*Pisum sativum* L.	Fabaceae	*Ascophyllum nodosum* extract (ANE) and chitosan suppressed pea powdery mildew by modulating Jasmonic acid and Salicylic acid-upregulated signaling pathways.	[84]
Pepper	*Capsicum annuum* L.	Solanaceae	Nano-chitosan positively affected plant morphogenesis, growth and physiology.	[134]
Potato	*Solanum tuberosum* L.	Solanaceae	Chitosan application may significantly increase root fresh and dry weight.	[135]
			Foliar spraying of chitosan combined with humic acid could lead to higher tuber yield and yield components.	[136]
			Chitosan (75 mg/L) and oligo-chitosan (50 mg/L) can increase plant growth and induce defense mechanisms for drought stress tolerance.	[137]
			Chitosan can inhibit the growth and spore germination and induce resistance against *Fusarium oxysporum*.	[138]
			Growth and spore germination of *Phytophthora infestans* were inhibited by chitosan.	[139]
Sweet potato	*Ipomoea batatas* L.	Convolvulaceae	Chitosan slowed down the cell growth, induced cell necrosis and significantly affected fatty acid composition of *Ceratocystis fimbriata*.	[140]
Tomato	*Solanum lycopersicum* L.	Solanaceae	Chitosan had positive effects on plant growth promotion and control of *Ralstonia solanacearum*.	[141]
			Foliar application of salicylic acid and chitosan at 75 mg L^−1^ may be utilized at early growth stage for getting maximum fruit yield in summer tomato.	[90]
			Chitosan ameliorated viral load, stimulated gas exchange and regulated *PAL5* expression, while it decreased the adverse impacts of *Cucumber mosaic virus* (CMV).	[142]
			Chitosan indicated the appropriate results to inhibit the infection caused by *Rhizopus stolonifer* on the tomato fruits.	[143]
			Chitosan combined with chelated copper had a higher efficiency in the enzyme activation associated with pathogenicity than chitosan or copper acting alone.	[94]
			Chitosan + compost + arbuscular mycorrhizal fungi application improved tomato growth.	[144]

**Table 2:** The effects of chitin on vegetable crops.

Plant	Scientific Name	Plant Family	Key Point	Reference
Cabbage and strawberry	*Brassica oleracea* cv. Shoshu and *Fragaria* × *ananassa*) var. Yotsuboshi	Brassicaceae and Rosaceae	Chitin nanofibers induced plant resistance against *Alternaria brassicicola Colletotrichum fructicola* and increased plant growth.	[186]
Cabbage		Brassicaceae	Chitin and *Trichoderma* formulation	[189]
			reduced the incidence of complex diseases *Sclerotinia sclerotiorum* and *Meloidogyne incognita.*	
Chili pepper	*Capsicum annum* L.	Solanaceae	Chitin and salicylic acid application along with antagonists (fluorescent pseudomonads SE21 and RD41) effectively controlled damping off (*Rhizoctonia solani*) of seedlings.	[85]
			Soil amendments with chitin effectively controlled *Meloidogyne javanica*) and *Fusarium solani* infections.	[192]
Eggplant	*Solanum melongena* L.	Solanaceae	Soil amendments with chitin obtained from crabs suppressed *Verticcilium* wilt in plants.	[193]
Lettuce	*Lactuca sativa* L.	Asteraceae	Peat supplemented with chitin increased the growth of lettuce plants and siderophore and chitinase genes.	[182]
			Soil application of chitin combined with foliar application of betaine improved crop performance under water stress conditions.	[190]
			The application of chitin-rich residues in growth medium increased lettuce plant growth and improved post-harvest quality.	[194]
Pigeon pea	*Cajanus cajan* L.	Fabaceae	Peat supplemented with chitin increased seedling emergence and growth of seedlings.	[181]
Tomato	*Solanum lycopersicum* L.	Solanaceae	Post-harvest treatment of tomato fruit with chitin induced resistance to *Botrytis cinerea* infections.	[180]
			Foliar application of chitin-based inoculum of *Paenibacillus elgii* HOA73 inhibited gray mold infections in fruit.	[183]
			Nanofiber complex of protein/CaCO3/chitin increased plant growth through efficient minerals release.	[184]
			Chitin nanofibers induced plant growth through the increased nitrogen use efficiency.	[188]
			Complexes of protein/CaCO3/chitin and protein/chitin nanofiber reduced *Fusarium* wilt incidence.	[187]
			Combined application of chitin and chitosan reduced the incidence of *Rhizoctonia solani*, *Fusarium solani* and *Sclerotium rolfsii* in plants.	[195,196]
			Chitin incorporation in the soil reduced root galls from nematode infections.	[197]

## Data Availability

The study did not report any data.

## References

[1] Baudoin W., Nono-Womdim R., Lutaladio N., Hodder A., Castilla N., Leonardi C., De Pascale S., Qaryouti M. (2013). Good Agricultural Practices for Greenhouse Vegetable Crops.

[2] Calvo P., Nelson L., Kloepper J.W. (2014). Agricultural uses of plant biostimulants. Plant Soil.

[3] Parađiković N., Teklić T., Zeljković S., Lisjak M., Špoljarević M. (2019). Biostimulants research in some horticultural plant species—A review. Food Energy Secur..

[4] Russo R.O., Berlyn G.P. (1991). The use of organic biostimulants to help low input sustainable agriculture. J. Sustain. Agric..

[5] Caradonia F., Battaglia V., Righi L., Pascali G., La Torre A. (2019). Plant Biostimulant Regulatory Framework: Prospects in Europe and Current Situation at International Level. J. Plant Growth Regul..

[6] Ekin Z. (2019). Integrated use of humic acid and plant growth promoting rhizobacteria to ensure higher potato productivity in sustainable agriculture. Sustainability.

[7] Kashyap P.L., Xiang X., Heiden P. (2015). Chitosan nanoparticle based delivery systems for sustainable agriculture. Int. J. Biol. Macromol..

[8] Shukla P.S., Mantin E.G., Adil M., Bajpai S., Critchley A.T., Prithiviraj B. (2019). *Ascophyllum nodosum*-based biostimulants: Sustainable applications in agriculture for the stimulation of plant growth, stress tolerance, and disease management. Front. Plant Sci..

[9] Abbas S.M. (2013). The influence of biostimulants on the growth and on the biochemical composition of *Vicia faba* CV. Giza 3 beans. Rom. Biotechnol. Lett..

[10] Gemin L.G., Mógor Á.F., De Oliveira Amatussi J., Mógor G. (2019). Microalgae associated to humic acid as a novel biostimulant improving onion growth and yield. Sci. Hortic..

[11] Dogan A., Erler F., Erkan M., Ates A.O., Sabanci H.S., Polat E. (2016). Microbial-based production system: A novel approach for plant growth and pest and disease management in greenhouse-grown peppers (*Capsicum annuum* L.). J. Agric. Sci. Technol..

[12] Olaetxea M., De Hita D., Garcia C.A., Fuentes M., Baigorri R., Mora V., Garnica M., Urrutia O., Erro J., Zamarreño A.M. (2018). Hypothetical framework integrating the main mechanisms involved in the promoting action of rhizospheric humic substances on plant root- and shoot- growth. Appl. Soil Ecol..

[13] Olivares F.L., Busato J.G., de Paula A.M., da Silva Lima L., Aguiar N.O., Canellas L.P. (2017). Plant growth promoting bacteria and humic substances: Crop promotion and mechanisms of action. Chem. Biol. Technol. Agric..

[14] Chakraborty M., Hasanuzzaman M., Rahman M., Khan M.A.R., Bhowmik P., Mahmud N.U., Tanveer M., Islam T. (2020). Mechanism of plant growth promotion and disease suppression by chitosan biopolymer. Agriculture.

[15] Wong W.S., Zhong H.T., Cross A.T., Wan J., Yong H. (2020). Plant Biostimulants in Vermicomposts: Characteristics and Plausible Mechanisms. Chem. Biol. Plant Biostimulants.

[16] De Saeger J., Van Praet S., Vereecke D., Park J., Jacques S., Han T., Depuydt S. (2020). Toward the molecular understanding of the action mechanism of *Ascophyllum nodosum* extracts on plants. J. Appl. Phycol..

[17] Galvão Í.M., dos Santos O.F., de Souza M.L.C., de Jesus Guimarães J., Kühn I.E., Broetto F. (2019). Biostimulants action in common bean crop submitted to water deficit. Agric. Water Manag..

[18] Petropoulos S.A., Fernandes Â., Plexida S., Chrysargyris A., Tzortzakis N., Barreira J., Barros L., Ferreira I.C. (2020). Biostimulants application alleviates water stress effects on yield and chemical composition of greenhouse green bean (*Phaseolus vulgaris* L.). Agronomy.

[19] Petropoulos S.A., Taofiq O., Fernandes Â., Tzortzakis N., Ciric A., Sokovic M., Barros L., Ferreira I.C. (2019). Bioactive properties of greenhouse-cultivated green beans *Phaseolus vulgaris* L.) under biostimulants and water-stress effect. J. Sci. Food Agric..

[20] Vurukonda S.S.K.P., Vardharajula S., Shrivastava M., SkZ A. (2016). Enhancement of drought stress tolerance in crops by plant growth promoting rhizobacteria. Microbiol. Res..

[21] Battacharyya D., Babgohari M.Z., Rathor P., Prithiviraj B. (2015). Seaweed extracts as biostimulants in horticulture. Sci. Hortic..

[22] Koleška I., Hasanagić D., Todorović V., Murtić S., Klokić I., Paradiković N., Kukavica B. (2017). Biostimulant prevents yield loss and reduces oxidative damage in tomato plants grown on reduced NPK nutrition. J. Plant Interact..

[23] Pereira C., Dias M.I., Petropoulos S.A., Plexida S., Chrysargyris A., Tzortzakis N., Calhelha R.C., Ivanov M., Stojković D., Soković M. (2019). The effects of biostimulants, biofertilizers and water-stress on nutritional value and chemical composition of two spinach genotypes (*Spinacia oleracea* L.). Molecules.

[24] Petropoulos S.A. (2020). Practical applications of plant biostimulants in greenhouse vegetable crop production. Agronomy.

[25] Canellas L.P., Olivares F.L., Aguiar N.O., Jones D.L., Nebbioso A., Mazzei P., Piccolo A. (2015). Humic and fulvic acids as biostimulants in horticulture. Sci. Hortic..

[26] Du Jardin P. (2015). Plant biostimulants: Definition, concept, main categories and regulation. Sci. Hortic..

[27] Colla G., Rouphael Y. (2015). Biostimulants in horticulture. Sci. Hortic..

[28] Kurepin L.V., Zaman M., Pharis R.P. (2014). Phytohormonal basis for the plant growth promoting action of naturally occurring biostimulators. J. Sci. Food Agric..

[29] Karkanis A., Ntatsi G., Alemardan A., Petropoulos S., Bilalis D. (2019). Interference of weeds in vegetable crop cultivation, in the changing climate of Southern Europe with emphasis on drought and elevated temperatures: A review. J. Agric. Sci..

[30] Iriti M., Vitalini S. (2020). Sustainable crop protection, global climate change, food security and safety—plant immunity at the crossroads. Vaccines.

[31] Toscano S., Romano D., Massa D., Bulgari R., Franzoni G., Ferrante A. (2018). Biostimulant applications in low input horticultural cultivation systems. Italus Hortus.

[32] De Pascale S., Rouphael Y., Colla G. (2018). Plant biostimulants: Innovative tool for enhancing plant nutrition in organic farming. Eur. J. Hortic. Sci..

[33] Rouphael Y., Colla G. (2020). Editorial: Biostimulants in Agriculture. Front. Plant Sci..

[34] Yadav M., Goswami P., Paritosh K., Kumar M., Pareek N., Vivekanand V. (2019). Seafood waste: A source for preparation of commercially employable chitin/chitosan materials. Bioresour. Bioprocess..

[35] Philibert T., Lee B.H., Fabien N. (2017). Current Status and New Perspectives on Chitin and Chitosan as Functional Biopolymers. Appl. Biochem. Biotechnol..

[36] Meramo-Hurtado S.I., González-Delgado Á.D. (2020). Application of Techno-economic and Sensitivity Analyses as Decision-Making Tools for Assessing Emerging Large-Scale Technologies for Production of Chitosan-Based Adsorbents. ACS Omega.

[37] Sharp R. (2013). A Review of the Applications of Chitin and Its Derivatives in Agriculture to Modify Plant-Microbial Interactions and Improve Crop Yields. Agronomy.

[38] Dutta P.K., Ravikumar M.N.V., Dutta J. (2002). Chitin and chitosan for versatile applications. J. Macromol. Sci. Polym. Rev..

[39] Ni Mhurchu C., Poppitt S.D., McGill A.T., Leahy F.E., Bennett D.A., Lin R.B., Ormrod D., Ward L., Strik C., Rodgers A. (2004). The effect of the dietary supplement, Chitosan, on body weight: A randomised controlled trial in 250 overweight and obese adults. Int. J. Obes..

[40] Liu X., Zhi X., Liu Y., Wu B., Sun Z., Shen J. (2012). Effect of chitosan, O-carboxymethyl chitosan, and N-[(2-hydroxy-3- N, N -dimethylhexadecyl ammonium)propyl] chitosan chloride on overweight and insulin resistance in a murine diet-induced obesity. J. Agric. Food Chem..

[41] Santas J., Espadaler J., Mancebo R., Rafecas M. (2012). Selective in vivo effect of chitosan on fatty acid, neutral sterol and bile acid excretion: A longitudinal study. Food Chem..

[42] Dash M., Chiellini F., Ottenbrite R.M., Chiellini E. (2011). Chitosan—A versatile semi-synthetic polymer in biomedical applications. Prog. Polym. Sci..

[43] Jayakumar R., Menon D., Manzoor K., Nair S.V., Tamura H. (2010). Biomedical applications of chitin and chitosan based nanomaterials—A short review. Carbohydr. Polym..

[44] Tahamtan A., Ghaemi A., Gorji A., Kalhor H.R., Sajadian A., Tabarraei A., Moradi A., Atyabi F., Kelishadi M. (2014). Antitumor effect of therapeutic HPV DNA vaccines with chitosan-based nanodelivery systems. J. Biomed. Sci..

[45] Garg T., Rath G., Goyal A.K. (2015). Biomaterials-based nanofiber scaffold: Targeted and controlled carrier for cell and drug delivery. J. Drug Target..

[46] Das S.N., Madhuprakash J., Sarma P.V.S.R.N., Purushotham P., Suma K., Manjeet K., Rambabu S., El Gueddari N.E., Moerschbacher B.M., Podile A.R. (2015). Biotechnological approaches for field applications of chitooligosaccharides (COS) to induce innate immunity in plants. Crit. Rev. Biotechnol..

[47] El Hadrami A., Adam L.R., El Hadrami I., Daayf F. (2010). Chitosan in plant protection. Mar. Drugs.

[48] Hemantaranjan A. (2014). A Future Perspective in Crop Protection: Chitosan and its Oligosaccharides. Adv. Plants Agric. Res..

[49] Nitschke J., Altenbach H.J., Malolepszy T., Mölleken H. (2011). A new method for the quantification of chitin and chitosan in edible mushrooms. Carbohydr. Res..

[50] Pires C.T.G.V.M.T., Vilela J.A.P., Airoldi C. (2014). The Effect of Chitin Alkaline Deacetylation at Different Condition on Particle Properties. Procedia Chem..

[51] Batista A.C.L., Melo T.B.L., Paiva W.S., Souza F.S.D., Campos-Takaki G.M. (2020). DE Economic microbiological conversion of agroindustrial wastes to fungi chitosan. An. Acad. Bras. Cienc..

[52] Amorim R.V.S., Ledingham W.M., Kennedy J.F., Campos-Takaki G.M. (2006). Chitosan from *Syncephalastrum racemosum* using sugar cane substrates as inexpensive carbon sources. Food Biotechnol..

[53] Kaczmarek M.B., Struszczyk-Swita K., Li X., Szczęsna-Antczak M., Daroch M. (2019). Enzymatic modifications of chitin, chitosan, and chitooligosaccharides. Front. Bioeng. Biotechnol..

[54] Younes I., Rinaudo M. (2015). Chitin and chitosan preparation from marine sources. Structure, properties and applications. Mar. Drugs.

[55] Gortari M.C., Hours R.A. (2013). Biotechnological processes for chitin recovery out of crustacean waste: A mini-review. Electron. J. Biotechnol..

[56] Kaur S., Dhillon G.S. (2015). Recent trends in biological extraction of chitin from marine shell wastes: A review. Crit. Rev. Biotechnol..

[57] Brar S.K., Dhillon G.S., Soccol C.R. (2014). Biotransformation of Waste Biomass into High Value Biochemicals.

[58] Sachindra N.M., Mahendrakar N.S. (2010). Stability of carotenoids recovered from shrimp waste and their use as colorant in fish sausage. J. Food Sci. Technol..

[59] Benhabiles M.S., Abdi N., Drouiche N., Lounici H., Pauss A., Goosen M.F.A., Mameri N. (2013). Protein recovery by ultrafiltration during isolation of chitin from shrimp shells *Parapenaeus longirostris*. Food Hydrocoll..

[60] Sachindra N.M., Bhaskar N., Mahendrakar N.S. (2005). Carotenoids in different body components of Indian shrimps. J. Sci. Food Agric..

[61] Cogollo-Herrera K., Bonfante-Álvarez H., De Ávila-Montiel G., Barros A.H., González-Delgado Á.D. (2018). Techno-economic sensitivity analysis of large scale chitosan production process from shrimp shell wastes. Chem. Eng. Trans..

[62] Bajaj M., Winter J., Gallert C. (2011). Effect of deproteination and deacetylation conditions on viscosity of chitin and chitosan extracted from *Crangon crangon* shrimp waste. Biochem. Eng. J..

[63] Peter M.G. (1995). Applications and Environmental Aspects of Chitin and Chitosan. J. Macromol. Sci. Part A.

[64] Hafsa J., Smach M.A., Ben Mrid R., Sobeh M., Majdoub H., Yasri A. (2021). Functional properties of chitosan derivatives obtained through Maillard reaction: A novel promising food preservative. Food Chem..

[65] Irastorza A., Zarandona I., Andonegi M., Guerrero P., de la Caba K. (2021). The versatility of collagen and chitosan: From food to biomedical applications. Food Hydrocoll..

[66] Rajabi M., McConnell M., Cabral J., Ali M.A. (2021). Chitosan hydrogels in 3D printing for biomedical applications. Carbohydr. Polym..

[67] Shoueir K.R., El-Desouky N., Rashad M.M., Ahmed M.K., Janowska I., El-Kemary M. (2021). Chitosan based-nanoparticles and nanocapsules: Overview, physicochemical features, applications of a nanofibrous scaffold, and bioprinting. Int. J. Biol. Macromol..

[68] Yanat M., Schroën K. (2021). Preparation methods and applications of chitosan nanoparticles; with an outlook toward reinforcement of biodegradable packaging. React. Funct. Polym..

[69] Abhinaya M., Parthiban R., Kumar P.S., Vo D.V.N. (2021). A review on cleaner strategies for extraction of chitosan and its application in toxic pollutant removal. Environ. Res..

[70] Saheed I.O., Da Oh W., Suah F.B.M. (2021). Chitosan modifications for adsorption of pollutants—A review. J. Hazard. Mater..

[71] Yu J., Wang D., Geetha N., Khawar K.M., Jogaiah S., Mujtaba M. (2021). Current trends and challenges in the synthesis and applications of chitosan-based nanocomposites for plants: A review. Carbohydr. Polym..

[72] Rinaudo M. (2006). Chitin and chitosan: Properties and applications. Prog. Polym. Sci..

[73] Mujtaba M., Khawar K.M., Camara M.C., Carvalho L.B., Fraceto L.F., Morsi R.E., Elsabee M.Z., Kaya M., Labidi J., Ullah H. (2020). Chitosan-based delivery systems for plants: A brief overview of recent advances and future directions. Int. J. Biol. Macromol..

[74] Salama A. (2021). Recent progress in preparation and applications of chitosan/calcium phosphate composite materials. Int. J. Biol. Macromol..

[75] Etemadi S., Soroush Barhaghi M.H., Leylabadlo H.E., Memar M.Y., Mohammadi A.B., Ghotaslou R. (2021). The synergistic effect of turmeric aqueous extract and chitosan against multiple drug-resistant bacteria. New Microbes New Infect..

[76] Malerba M., Cerana R. (2018). Recent advances of chitosan applications in plants. Polymers.

[77] Choi C., Nam J.P., Nah J.W. (2016). Application of chitosan and chitosan derivatives as biomaterials. J. Ind. Eng. Chem..

[78] Bellich B., D’Agostino I., Semeraro S., Gamini A., Cesàro A. (2016). “The good, the Bad and the Ugly” of Chitosans. Mar. Drugs.

[79] Malerba M., Cerana R. (2016). Chitosan effects on plant systems. Int. J. Mol. Sci..

[80] Pichyangkura R., Chadchawan S. (2015). Biostimulant activity of chitosan in horticulture. Sci. Hortic..

[81] El Amerany F., Meddich A., Wahbi S., Porzel A., Taourirte M., Rhazi M., Hause B. (2020). Foliar application of chitosan increases tomato growth and influences mycorrhization and expression of endochitinase-encoding genes. Int. J. Mol. Sci..

[82] Mondal M.M.A., Puteh A.B., Dafader N.C., Rafii M.Y., Malek M.A. (2013). Foliar application of chitosan improves growth and yield in maize. J. Food Agric. Environ..

[83] Younes I., Sellimi S., Rinaudo M., Jellouli K., Nasri M. (2014). Influence of acetylation degree and molecular weight of homogeneous chitosans on antibacterial and antifungal activities. Int. J. Food Microbiol..

[84] Patel J.S., Selvaraj V., Gunupuru L.R., Rathor P.K., Prithiviraj B. (2020). Combined application of *Ascophyllum nodosum* extract and chitosan synergistically activates host-defense of peas against powdery mildew. BMC Plant Biol..

[85] Rajkumar M., Lee K.J., Freitas H. (2008). Effects of chitin and salicylic acid on biological control activity of *Pseudomonas* spp. against damping off of pepper. S. Afr. J. Bot..

[86] Doares S.H., Syrovets T., Weiler E.W., Ryan C.A. (1995). Oligogalacturonides and chitosan activate plant defensive genes through the octadecanoid pathway. Proc. Natl. Acad. Sci. USA.

[87] Bell A.A., Hubbard J.C., Liu L., Michael Davis R., Subbarao K.V. (1998). Effects of chitin and chitosan on the incidence and severity of Fusarium yellows of celery. Plant Dis..

[88] González L.M., Guerrero Y.R., Rodríguez A.F., Vázquez M.N. (2015). Effect of Seed Treatment With Chitosan On The Growth Of Rice (*Oryza Sativa* L.) Seedlings cv. Inca LP-5 In Saline Medium. Cultiv. Trop..

[89] Pandey P., Kumar Verma M., De N. (2018). Chitosan in Agricultural Context-A Review. Bull. Environ. Pharmacol. Life Sci.

[90] Mondal M.M.A., Puteh A.B., Dafader N.C. (2016). Foliar application of chitosan improved morpho-physiological attributes and yield in summer tomato (*Solanum lycopersicum*). Pak. J. Agric. Sci..

[91] Bittelli M., Flury M., Campbell G.S., Nichols E.J. (2001). Reduction of transpiration through foliar application of chitosan. Agric. For. Meteorol..

[92] Ghasemi Pirbalouti A., Malekpoor F., Salimi A., Golparvar A. (2017). Exogenous application of chitosan on biochemical and physiological characteristics, phenolic content and antioxidant activity of two species of basil (*Ocimum ciliatum* and *Ocimum basilicum*) under reduced irrigation. Sci. Hortic..

[93] Benhamou N., Thériault G. (1992). Treatment with chitosan enhances resistance of tomato plants to the crown and root rot pathogen *Fusarium oxysporum* f. sp. *radicis-lycopersici*. Physiol. Mol. Plant Pathol..

[94] Adamuchio-Oliveira L.G., Mazaro S.M., Mógor G., Sant’Anna-Santos B.F., Mógor Á.F. (2020). Chitosan associated with chelated copper applied on tomatoes: Enzymatic and anatomical changes related to plant defense responses. Sci. Hortic..

[95] Xu C., Mou B. (2018). Chitosan as soil amendment affects lettuce growth, photochemical efficiency, and gas exchange. Horttechnology.

[96] Parvin M.A., Zakir H.M., Sultana N., Kafi A., Seal H.P. (2019). Effects of different application methods of chitosan on growth, yield and quality of tomato (*Lycopersicon esculentum* Mill.). Arch. Agric. Environ. Sci..

[97] Hidangmayum A., Dwivedi P., Katiyar D., Hemantaranjan A. (2019). Application of chitosan on plant responses with special reference to abiotic stress. Physiol. Mol. Biol. Plants.

[98] Hataf N., Ghadir P., Ranjbar N. (2018). Investigation of soil stabilization using chitosan biopolymer. J. Clean. Prod..

[99] Wan M.W., Petrisor I.G., Lai H.T., Kim D., Yen T.F. (2004). Copper adsorption through chitosan immobilized on sand to demonstrate the feasibility for in situ soil decontamination. Carbohydr. Polym..

[100] Benhamou N., Lafontaine P., Nicole M. (1994). Induction of systemic resistance to *Fusarium* crown and root rot in tomato plants by seed treatment with chitosan. Phyopathology.

[101] Jabeen N., Ahmad R. (2013). The activity of antioxidant enzymes in response to salt stress in safflower (*Carthamus tinctorius* L.) and sunflower (*Helianthus annuus* L.) seedlings raised from seed treated with chitosan. J. Sci. Food Agric..

[102] Jogaiah S., Satapute P., De Britto S., Konappa N., Udayashankar A.C. (2020). Exogenous priming of chitosan induces upregulation of phytohormones and resistance against cucumber powdery mildew disease is correlated with localized biosynthesis of defense enzymes. Int. J. Biol. Macromol..

[103] Zeng D., Luo X. (2012). Physiological Effects of Chitosan Coating on Wheat Growth and Activities of Protective Enzyme with Drought Tolerance. Open J. Soil Sci..

[104] Abdel-Aziz H. (2019). Effect of Priming with Chitosan Nanoparticles on Germination, Seedling Growth and Antioxidant Enzymes of Broad Beans. Catrina Int. J. Environ. Sci..

[105] Muley A.B., Singhal R.S. (2020). Extension of postharvest shelf life of strawberries (*Fragaria ananassa*) using a coating of chitosan-whey protein isolate conjugate. Food Chem..

[106] Madureira A.R., Pereira A., Castro P.M., Pintado M. (2015). Production of antimicrobial chitosan nanoparticles against food pathogens. J. Food Eng..

[107] Pavinatto A., de Almeida Mattos A.V., Malpass A.C.G., Okura M.H., Balogh D.T., Sanfelice R.C. (2020). Coating with chitosan-based edible films for mechanical/biological protection of strawberries. Int. J. Biol. Macromol..

[108] Jianglian D. (2013). Application of Chitosan Based Coating in Fruit and Vegetable Preservation: A Review. J. Food Process. Technol..

[109] Chen W., Jin T.Z., Gurtler J.B., Geveke D.J., Fan X. (2012). Inactivation of *Salmonella* on whole cantaloupe by application of an antimicrobial coating containing chitosan and allyl isothiocyanate. Int. J. Food Microbiol..

[110] Kumaraswamy R.V., Saharan V., Kumari S., Chandra Choudhary R., Pal A., Sharma S.S., Rakshit S., Raliya R., Biswas P. (2021). Chitosan-silicon nanofertilizer to enhance plant growth and yield in maize (*Zea mays* L.). Plant Physiol. Biochem..

[111] Cota-Arriola O., Onofre Cortez-Rocha M., Burgos-Hernández A., Marina Ezquerra-Brauer J., Plascencia-Jatomea M. (2013). Controlled release matrices and micro/nanoparticles of chitosan with antimicrobial potential: Development of new strategies for microbial control in agriculture. J. Sci. Food Agric..

[112] Vanti G.L., Masaphy S., Kurjogi M., Chakrasali S., Nargund V.B. (2020). Synthesis and application of chitosan-copper nanoparticles on damping off causing plant pathogenic fungi. Int. J. Biol. Macromol..

[113] Geries L., Omnia, Hashem S.M., Marey R.A. (2020). Soaking and foliar application with chitosan and nano chitosan to enhancing growth, productivity and quality of onion crop. Plant Arch..

[114] Sathiyaseelan A., Saravanakumar K., Mariadoss A.V.A., Ramachandran C., Hu X., Oh D.H., Wang M.H. (2021). Chitosan-tea tree oil nanoemulsion and calcium chloride tailored edible coating increase the shelf life of fresh cut red bell pepper. Prog. Org. Coat..

[115] Sonin D., Pochkaeva E., Zhuravskii S., Postnov V., Korolev D., Vasina L., Kostina D., Mukhametdinova D., Zelinskaya I., Skorik Y. (2020). Biological safety and biodistribution of chitosan nanoparticles. Nanomaterials.

[116] Mohammed M.A., Syeda J.T.M., Wasan K.M., Wasan E.K. (2017). An overview of chitosan nanoparticles and its application in non-parenteral drug delivery. Pharmaceutics.

[117] Ziani K., Ursúa B., Maté J.I. (2010). Application of bioactive coatings based on chitosan for artichoke seed protection. Crop Prot..

[118] Hawrylak-Nowak B., Dresler S., Rubinowska K., Matraszek-Gawron R. (2021). Eliciting effect of foliar application of chitosan lactate on the phytochemical properties of *Ocimum basilicum* L. and *Melissa officinalis* L.. Food Chem..

[119] Ibrahim E.A., Ramadan W.A. (2015). Effect of zinc foliar spray alone and combined with humic acid or/and chitosan on growth, nutrient elements content and yield of dry bean (*Phaseolus vulgaris* L.) plants sown at different dates. Sci. Hortic..

[120] Pereira A.E.S., Silva P.M., Oliveira J.L., Oliveira H.C., Fraceto L.F. (2017). Chitosan nanoparticles as carrier systems for the plant growth hormone gibberellic acid. Colloids Surfaces B Biointerfaces.

[121] Hu X., Saravanakumar K., Sathiyaseelan A., Wang M.H. (2020). Chitosan nanoparticles as edible surface coating agent to preserve the fresh-cut bell pepper (*Capsicum annuum* L. var. *grossum* (L.) Sendt). Int. J. Biol. Macromol..

[122] Samarah N.H., AL-Quraan N.A., Massad R.S., Welbaum G.E. (2020). Treatment of bell pepper (*Capsicum annuum* L.) seeds with chitosan increases chitinase and glucanase activities and enhances emergence in a standard cold test. Sci. Hortic..

[123] Kaur P., Duhan J.S., Thakur R. (2018). Comparative pot studies of chitosan and chitosan-metal nanocomposites as nano-agrochemicals against fusarium wilt of chickpea (*Cicer arietinum* L.). Biocatal. Agric. Biotechnol..

[124] Muthukrishnan S., Murugan I., Selvaraj M. (2019). Chitosan nanoparticles loaded with thiamine stimulate growth and enhances protection against wilt disease in Chickpea. Carbohydr. Polym..

[125] Akter J., Jannat R., Hossain M.M., Ahmed J.U., Rubayet M.T. (2018). Chitosan for Plant Growth Promotion and Disease Suppression against Anthracnose in Chilli. Int. J. Environ. Agric. Biotechnol..

[126] Zohara F., Surovy M.Z., Khatun A., Prince M.F.R.K., Akanda M.A.M., Rahman M., Islam M.T. (2019). Chitosan biostimulant controls infection of cucumber by *Phytophthora capsici* through suppression of asexual reproduction of the pathogen. Acta Agrobot..

[127] Attia M.S., El-Sayyad G.S., Abd Elkodous M., Khalil W.F., Nofel M.M., Abdelaziz A.M., Farghali A.A., El-Batal A.I., El Rouby W.M.A. (2021). Chitosan and EDTA conjugated graphene oxide antinematodes in Eggplant: Toward improving plant immune response. Int. J. Biol. Macromol..

[128] Sofy A.R., Hmed A.A., Alnaggar A.E.A.M., Dawoud R.A., Elshaarawy R.F.M., Sofy M.R. (2020). Mitigating effects of Bean yellow mosaic virus infection in faba bean using new carboxymethyl chitosan-titania nanobiocomposites. Int. J. Biol. Macromol..

[129] Liu Y., Wisniewski M., Kennedy J.F., Jiang Y., Tang J., Liu J. (2016). Chitosan and oligochitosan enhance ginger (*Zingiber officinale* Roscoe) resistance to rhizome rot caused by *Fusarium oxysporum* in storage. Carbohydr. Polym..

[130] Turan V. (2019). Confident performance of chitosan and pistachio shell biochar on reducing Ni bioavailability in soil and plant plus improved the soil enzymatic activities, antioxidant defense system and nutritional quality of lettuce. Ecotoxicol. Environ. Saf..

[131] Valderrama N.A., Jacinto H.C., Lay J., Flores E.Y., Zavaleta C.D., Delfín A.R. (2020). Factorial design for preparing chitosan nanoparticles and its use for loading and controlled release of indole-3-acetic acid with effect on hydroponic lettuce crops. Biocatal. Agric. Biotechnol..

[132] Abd El-Aziz M.E., Morsi S.M.M., Salama D.M., Abdel-Aziz M.S., Abd Elwahed M.S., Shaaban E.A., Youssef A.M. (2019). Preparation and characterization of chitosan/polyacrylic acid/copper nanocomposites and their impact on onion production. Int. J. Biol. Macromol..

[133] Mondal M.M.A., Malek M.A., Puteh A.B., Ismail M.R., Ashrafuzzaman M., Naher L. (2012). Effect of foliar application of chitosan on growth and yield in okra. Aust. J. Crop Sci..

[134] Asgari-Targhi G., Iranbakhsh A., Ardebili Z.O. (2018). Potential benefits and phytotoxicity of bulk and nano-chitosan on the growth, morphogenesis, physiology, and micropropagation of *Capsicum annuum*. Plant Physiol. Biochem..

[135] Asghari-Zakaria R., Maleki-Zanjani B., Sedghi E. (2009). Effect of in vitro chitosan application on growth and minituber yield of *Solanum tuberosum* L.. Plant Soil Environ..

[136] Harfoush E., Abdel-Razzek A., El-Adgham F., El-Sharkawy A. (2017). Effects of Humic Acid and Chitosan under Different Levels of Nitrogen and Potassium fertilizers on Growth and Yield potential of Potato plants (*Solanum tuberosum*, L.). Alexandria J. Agric. Sci..

[137] Muley A.B., Shingote P.R., Patil A.P., Dalvi S.G., Suprasanna P. (2019). Gamma radiation degradation of chitosan for application in growth promotion and induction of stress tolerance in potato (*Solanum tuberosum* L.). Carbohydr. Polym..

[138] Ren J., Tong J., Li P., Huang X., Dong P., Ren M. (2021). Chitosan is an effective inhibitor against potato dry rot caused by *Fusarium oxysporum*. Physiol. Mol. Plant Pathol..

[139] Huang X., You Z., Luo Y., Yang C., Ren J., Liu Y., Wei G., Dong P., Ren M. (2021). Antifungal activity of chitosan against *Phytophthora infestans*, the pathogen of potato late blight. Int. J. Biol. Macromol..

[140] Xing K., Li T.J., Liu Y.F., Zhang J., Zhang Y., Shen X.Q., Li X.Y., Miao X.M., Feng Z.Z., Peng X. (2018). Antifungal and eliciting properties of chitosan against *Ceratocystis fimbriata* in sweet potato. Food Chem..

[141] Algam S.A.E., Xie G., Li B., Yu S., Su T., Larsen J. (2010). Effects of *Paenibacillus* strains and chitosan on plant growth promotion and control of Ralstonia wilt in tomato. J. Plant Pathol..

[142] Rendina N., Nuzzaci M., Scopa A., Cuypers A., Sofo A. (2019). Chitosan-elicited defense responses in Cucumber mosaic virus (CMV)-infected tomato plants. J. Plant Physiol..

[143] Hernández-Herrera R.M., Santacruz-Ruvalcaba F., Zañudo-Hernández J., Hernández-Carmona G. (2016). Activity of seaweed extracts and polysaccharide-enriched extracts from *Ulva lactuca* and *Padina gymnospora* as growth promoters of tomato and mung bean plants. J. Appl. Phycol..

[144] El Amerany F., Rhazi M., Wahbi S., Taourirte M., Meddich A. (2020). The effect of chitosan, arbuscular mycorrhizal fungi, and compost applied individually or in combination on growth, nutrient uptake, and stem anatomy of tomato. Sci. Hortic..

[145] Feng J., Zhao L., Yu Q. (2004). Receptor-mediated stimulatory effect of oligochitosan in macrophages. Biochem. Biophys. Res. Commun..

[146] Mao S., Shuai X., Unger F., Simon M., Bi D., Kissel T. (2004). The depolymerization of chitosan: Effects on physicochemical and biological properties. Int. J. Pharm..

[147] Kuroiwa T., Ichikawa S., Hiruta O., Sato S., Mukataka S. (2002). Factors affecting the composition of oligosaccharides produced in chitosan hydrolysis using immobilized chitosanases. Biotechnol. Prog..

[148] Darvill A., Augur C., Bergmann C., Carlson R.W., Cheong J.-J., Eberhard S., Hahn M.G., Ló V.-M., Marfa V., Meyer B. (1992). Oligosaccharins—oligosaccharides that regulate growth, development and defence responses in plants. Glycobiology.

[149] Xia W., Liu P., Zhang J., Chen J. (2011). Biological activities of chitosan and chitooligosaccharides. Food Hydrocoll..

[150] Zhang B., Ramonell K., Somerville S., Stacey G. (2002). Characterization of early, chitin-induced gene expression in Arabidopsis. Mol. Plant-Microbe Interact..

[151] Yin H., Zhao X., Du Y. (2010). Oligochitosan: A plant diseases vaccine—A review. Carbohydr. Polym..

[152] Qing M., Hui S., Yuguang D., Xiaoming Z., Hongsheng S. (2004). Induction of oligosaccharide to ultrastructure of cucumber resistance to powdery mildew fungus. Zhi Wu Bing Li Xue Bao Acta Phytopathol. Sin..

[153] Xu J., Zhao X., Han X., Du Y. (2007). Antifungal activity of oligochitosan against Phytophthora capsici and other plant pathogenic fungi in vitro. Pestic. Biochem. Physiol..

[154] Nguyen V.T., Tran K.V.Q., Tran Q.N. (2018). Effect of oligochitosan-coated silver nanoparticles (OCAgNPs) on the growth and reproduction of three species *Phytophthora* in vitro. Arch. Phytopathol. Plant Prot..

[155] Moenne A., González A. (2021). Chitosan-, alginate- carrageenan-derived oligosaccharides stimulate defense against biotic and abiotic stresses, and growth in plants: A historical perspective. Carbohydr. Res..

[156] Sultana S., Islam M., Khatun M.A., Hassain M.A., Huque R. (2017). Effect of Foliar Application of Oligo-chitosan on Growth, Yield and Quality of Tomato and Eggplant. Asian J. Agric. Res..

[157] Dzung P.D., Van Phu D., Du B.D., Ngoc L.S., Duy N.N., Hiet H.D., Nghia D.H., Thang N.T., Van Le B., Hien N.Q. (2017). Effect of foliar application of oligochitosan with different molecular weight on growth promotion and fruit yield enhancement of chili plant. Plant Prod. Sci..

[158] Sultana S., Dafader N.C., Kabir H., Khatun F., Rahman M., Alam J. (2015). Application of Oligo-Chitosan in Leaf Vegetable (Spinach) Production. Nucl. Sci. Appl..

[159] Sun G., Yang Q., Zhang A., Guo J., Liu X., Wang Y., Ma Q. (2018). Synergistic effect of the combined bio-fungicides ε-poly-L-lysine and chitooligosaccharide in controlling grey mould (*Botrytis cinerea*) in tomatoes. Int. J. Food Microbiol..

[160] Li Y., Yin H., Wang Q., Zhao X., Du Y., Li F. (2009). Oligochitosan induced Brassica napus L. production of NO and H2O2 and their physiological function. Carbohydr. Polym..

[161] Rakpenthai A., Khaksar G., Burow M., Olsen C.E., Sirikantaramas S. (2019). Metabolic changes and increased levels of bioactive compounds in white radish (*Raphanus sativus* L. Cv. 01) sprouts elicited by oligochitosan. Agronomy.

[162] Kurita K. (2006). Chitin and chitosan: Functional biopolymers from marine crustaceans. Mar. Biotechnol..

[163] Crestini C., Kovac B., Giovannozzi-Sermanni G. (1996). Production and isolation of chitosan by submerged and solid-state fermentation from *Lentinus edodes*. Biotechnol. Bioeng..

[164] Wu T., Zivanovic S., Draughon F.A., Sams C.E. (2004). Chitin and chitosan-value-added products from mushroom waste. J. Agric. Food Chem..

[165] Rogg L.E., Fortwendel J.R., Juvvadi P.R., Steinbach W.J. (2012). Regulation of expression, activity and localization of fungal chitin synthases. Med. Mycol..

[166] Pusztahelyi T. (2018). Chitin and chitin-related compounds in plant–fungal interactions. Mycology.

[167] Ramírez M.Á., Rodríguez A.T., Alfonso L., Peniche C. (2010). Chitin and its derivatives as biopolymers with potetial agricultural applications. Biotecnol. Appl..

[168] Chitin Market, 2017 Chitin Market: Agrochemical End Use Industry Segment Inclined Towards High Growth—Moderate Value during the Forecast Period: Global Industry Analysis (2012–2016) and Opportunity Assessment (2017–2027). https://www.futuremarketinsights.com/reports/chitin-market.

[169] Vigil M., Laza M.P., Moran-Palacios H., Cabal J.V.A. (2020). Optimizing the environmental profile of fresh-cut produce: Life cycle assessment of novel decontamination and sanitation techniques. Sustainability.

[170] Kumar S., Mukherjee A., Dutta J. (2020). Chitosan based nanocomposite films and coatings: Emerging antimicrobial food packaging alternatives. Trends Food Sci. Technol..

[171] Tong S., Sophal L., Samoeun B., Buntong B., Kong T., Acedo A.L. Chitosan extraction from seafood waste and its potential application in production and postharvest horticulture. Proceedings of the Acta Horticulturae, International Society for Horticultural Science (ISHS).

[172] Wan J., Zhang X.C., Stacey G. (2008). Chitin signaling and plant disease resistance. Plant Signal. Behav..

[173] Eckardt N.A. (2008). Chitin signaling in plants: Insights into the perception of fungal pathogens and rhizobacterial symbionts. Plant Cell.

[174] De Boer W., Gerards S., Klein Gunnewiek P.J.A., Modderman R. (1999). Response of the chitinolytic microbial community to chitin amendments of dune soils. Biol. Fertil. Soils.

[175] Brown J., Neville F., Sarathchandra S., Watson R., Cox N. Effects of chitin amendment on plant growth, microbial populations and nematodes in soil. Proceedings of the 48th New Zealand Plant Protection Conference.

[176] Li Y., Chen X.G., Liu N., Liu C.S., Liu C.G., Meng X.H., Yu L.J., Kenendy J.F. (2007). Physicochemical characterization and antibacterial property of chitosan acetates. Carbohydr. Polym..

[177] Sarathchandra S.U., Watson R.N., Cox N.R., Di Menna M.E., Brown J.A., Burch G., Neville F.J. (1996). Effects of chitin amendment of soil on microorganisms, nematodes, and growth of white clover (*Trifolium repens* L.) and perennial ryegrass (*Lolium perenne* L.). Biol. Fertil. Soils.

[178] Dhall R.K. (2013). Advances in Edible Coatings for Fresh Fruits and Vegetables: A Review. Crit. Rev. Food Sci. Nutr..

[179] Cho Y.I., No H.K., Meyers S.P. (1998). Physicochemical Characteristics and Functional Properties of Various Commercial Chitin and Chitosan Products. J. Agric. Food Chem..

[180] Sun C., Fu D., Jin L., Chen M., Zheng X., Yu T. (2018). Chitin isolated from yeast cell wall induces the resistance of tomato fruit to *Botrytis cinerea*. Carbohydr. Polym..

[181] Manjula K., Podile A.R. (2005). Increase in seedling emergence and dry weight of pigeon pea in the field with chitin-supplemented formulations of *Bacillus subtilis* AF 1. World J. Microbiol. Biotechnol..

[182] De Tender C., Mesuere B., Van der Jeugt F., Haegeman A., Ruttink T., Vandecasteele B., Dawyndt P., Debode J., Kuramae E.E. (2019). Peat substrate amended with chitin modulates the N-cycle, siderophore and chitinase responses in the lettuce rhizobiome. Sci. Rep..

[183] Kim Y.C., Hur J.Y., Park S.K. (2019). Biocontrol of *Botrytis cinerea* by chitin-based cultures of Paenibacillus elgii HOA73. Eur. J. Plant Pathol..

[184] Aklog Y.F., Egusa M., Kaminaka H., Izawa H., Morimoto M., Saimoto H., Ifuku S. (2016). Protein/CaCo3/Chitin nanofiber complex prepared from crab shells by simple mechanical treatment and its effect on plant growth. Int. J. Mol. Sci..

[185] Egusa M., Matsui H., Urakami T., Okuda S., Ifuku S., Nakagami H., Kaminaka H. (2015). Chitin nanofiber elucidates the elicitor activity of polymeric chitin in plants. Front. Plant Sci..

[186] Parada R.Y., Egusa M., Aklog Y.F., Miura C., Ifuku S., Kaminaka H. (2018). Optimization of nanofibrillation degree of chitin for induction of plant disease resistance: Elicitor activity and systemic resistance induced by chitin nanofiber in cabbage and strawberry. Int. J. Biol. Macromol..

[187] Egusa M., Parada R.Y., Aklog Y.F., Ifuku S., Kaminaka H. (2019). Nanofibrillation enhances the protective effect of crab shells against *Fusarium* wilt disease in tomato. Int. J. Biol. Macromol..

[188] Egusa M., Matsukawa S., Miura C., Nakatani S., Yamada J., Endo T., Ifuku S., Kaminaka H. (2020). Improving nitrogen uptake efficiency by chitin nanofiber promotes growth in tomato. Int. J. Biol. Macromol..

[189] Loganathan M., Sible G.V., Maruthasalam S., Saravanakumar D., Raguchander T., Sivakumar M., Samiyappan R. (2010). *Trichoderma* and chitin mixture based bioformulation for the management of head rot (*Sclerotinia sclerotiorum* (Lib.) deBary)-root-knot (*Meloidogyne incognita* Kofoid and White; Chitwood) complex diseases of cabbage. Arch. Phytopathol. Plant Prot..

[190] Lin F.W., Lin K.H., Wu C.W., Chang Y.S. (2020). Effects of betaine and chitin on water use efficiency in lettuce (lactuca sativa var. capitata). HortScience.

[191] Fan Z., Qin Y., Liu S., Xing R., Yu H., Chen X., Li K., Li R., Wang X., Li P. (2019). The bioactivity of new chitin oligosaccharide dithiocarbamate derivatives evaluated against nematode disease (*Meloidogyne incognita*). Carbohydr. Polym..

[192] Hussain F., Shaukat S.S., Abid M., Usman F., Akbar M. (2013). Control of *Meloidogyne javanica* and *Fusarium solani* in chilli (*Capsicum annuum* L.) with the application of chitin. Pak. J. Nematol..

[193] Inderbitzin P., Ward J., Barbella A., Solares N., Izyumin D., Burman P., Chellemi D.O., Subbarao K.V. (2018). Soil microbiomes associated with verticillium wilt-suppressive broccoli and chitin amendments are enriched with potential biocontrol agents. Phytopathology.

[194] Muymas P., Pichyangkura R., Wiriyakitnateekul W., Wangsomboondee T., Chadchawan S., Seraypheap K. (2015). Effects of chitin-rich residues on growth and postharvest quality of lettuce. Biol. Agric. Hortic..

[195] Abd-El-Kareem F., El-Mougy N.S., El-Gamal N.G., Fotouh Y. (2006). Use of Chitin and Chitosan Against Tomato Root Rot Disease under Greenhouse Conditions. Res. J. Agric. Biol. Sci..

[196] El-Mougy N.S., El-Gamal N.G., Fotouh Y.O., Abd-El-Kareem F. (2006). Evaluation of Different Application Methods of Chitin and Chitosan for Controlling Tomato Root Rot Disease under Greenhouse and Field Conditions. Res. J. Agric. Biol. Sci..

[197] Radwan M.A., Farrag S.A.A., Abu-Elamayem M.M., Ahmed N.S. (2012). Extraction, characterization, and nematicidal activity of chitin and chitosan derived from shrimp shell wastes. Biol. Fertil. Soils.

